# Elder Abuse and Neglect: Training First Responders in Rural Arkansas to Recognize, Respond, and Report

**DOI:** 10.1093/geroni/igaa057.151

**Published:** 2020-12-16

**Authors:** Robin McAtee, Valerie Claar, Laura Spradley, Whitney Thomasson

**Affiliations:** 1 UAMS, Little Rock, Arkansas, United States; 2 Oaklawn Center on Aging, Hot Springs, Arkansas, United States; 3 University of Arkansas for Medical Sciences, Little Rock, Arkansas, United States

## Abstract

As the incidence of elder abuse and neglect continue to rise and plague our country’s older adults, it is imperative that their plight is recognized, reported and elicits an appropriate response. At least 1 out of 10 older adults suffer from at least one type of abuse each year (DOJ, 2020) and only 1 in 24 cases of elder abuse is ever reported to authorities (National Center on Elder Abuse, 2019). Since 41% of Arkansas’ population live in rural areas, reaching and educating first responders who work in these areas is a priority, yet has been a challenge. It has been ascertained that virtually no elder abuse or neglect related training for first responders occurs in Arkansas. In 2015, the Arkansas Geriatric Education Collaborative (a HRSA Geriatric Workforce Enhancement Program) developed an education program and mobilized it to multiple first responder groups including the AR State Police, multiple city and county paramedics’ organizations, EMTs, local police officers and fire fighters. The program was further enhance late in 2019 when the training was made available on-line in conjunction with dementia training. The content and methods of training and test results revealing knowledge gained will be reviewed. Follow-up stories from first responders who have put their training into action in the field will be told as they reveal how they have used their training to identify potential abuse, neglect and self-neglect cases and how they have recognized, reported and addressed specific cases.

